# The relationship between vaginal bleeding in the first and second trimester of pregnancy and preterm labor

**Published:** 2013-05

**Authors:** Seyedeh Hajar Sharami, Roya Faraji Darkhaneh, Ziba Zahiri, Forozan Milani, Maryam Asgharnia, Maryam Shakiba, Zirak Didar

**Affiliations:** 1 *Reproductive Health Research Center, Department of Obstetrics and Gynecology, Guilan University of Medical Sciences, Rasht, Iran.*; 2 *Guilan University of Medical Sciences, Rasht, Iran.*

**Keywords:** *Pregnancy*, *Preterm labor*, *Vaginal bleeding*

## Abstract

**Background: **Vaginal bleeding is a common complication during pregnancy, which is observed in about 1/4 of pregnancies and in half of cases can lead to abortion. If vaginal bleeding happens during pregnancy some adverse pregnancy outcomes, including perinatal mortality and morbidity, low birth weight and preterm delivery will be increased.

**Objective:** The aim of this study was to determine the relationship between vaginal bleeding and its characteristics in the first and second trimester of pregnancy and preterm labor.

**Materials and Methods: **This is a case-control study conducted on 440 pregnant women referred to Al-Zahra Hospital in Rasht, Iran. Data were collected by a form. The form included demographic characteristics and confounding factors, the occurrence of bleeding during pregnancy and its features. Data were analyzed by T test, chi square and logistic regression in SPSS 16.

**Results:** Findings showed that vaginal bleeding was associated with 3 times increased risk of preterm delivery (OR: 3, 1.84-4.89). Also, findings showed that bleeding characteristics including bleeding time, frequency, severity and intensity was significantly associated with preterm labor.

**Conclusion:** According to significant association between vaginal bleeding and preterm delivery, it seems that performing some interventions to prevent preterm labor could be appropriate.

## Introduction

Preterm delivery is the delivery before 37 weeks of gestation, which involves approximately 12% of all pregnancies. It is a main determinant of perinatal morbidity and mortality and could increase the risk of neurologic complications (1, 2). Vaginal bleeding affects up to 25% of all pregnancies, and in half of cases can lead to miscarriage (3). If vaginal bleeding happens during pregnancy, some adverse outcomes including mortality before and after birth, low birth weight and preterm delivery will be increased. As previously mentioned, vaginal bleeding is associated with two fold increased risk of preterm delivery. 

However, in spite of vaginal bleeding occurrence during pregnancy, yet about half of them have unknown causes (4). Because in many cases of bleeding, cause and source were unknown, therefore the possible mechanism of preterm labor followed by bleeding is still unclear (5).

Furthermore, a theory demonstrated that bleeding which followed by thrombin production can cause a proteolytic cascade which leads to fetal membranes destruction and finally premature rupture of membrance (PROM) (6). It has been determined that Thrombin, can stimulates uterine contractions and lead to premature labor without rupture of membrane (ROM). Based on another theory, bleeding can be a sign of subclinical uterine infection which could stimulate process of preterm labor (7). 

However, there are insufficient investigations related to probable mechanisms and characteristics of vaginal bleeding in preterm labor such as trimester of bleeding occurrence (1^st^ or 2^nd^), duration and severity, volume and bleeding episodes (4, 8). Therefore, according to contradictory and limited results, the aim of this research is to determine the relationship between vaginal bleeding and its characteristics in the first and second trimester of pregnancy and preterm labor in pregnant women.

## Materials and methods

This is a retrospective case-control study, which was conducted on 440 pregnant women referred to Alzahra Hospital from January 2010 to January 2012. We entered all cases that were eligible for inclusion in the study by convenience sampling. 

Enrolled participants were divided in two groups regarding to gestational age. Case group were women under 37 weeks and control group were women with more than 37 weeks of gestation age. The inclusion criteria were indicated more than 18 years of age, singleton pregnancy and having reliable gestational age. Exclusion criteria included iatrogenic preterm delivery, diabetes mellitus, hypertension, preeclampsia, placenta previa and abruption. The study was approved by the ethics committee of Guilan University of Medical Science.

Gestational age was obtained based on the last menstrual period (LMP) and confirmed by ultrasound examination prior to 20 weeks of gestation. Therefore, if the difference between LMP and ultrasound dating were within 10 days, LMP date was used to assign gestational age and if these two differed more than 10 days, the ultrasound date was considered as gestational age. Informed consent was obtained and enrolled participants took part in an interview with trained personnel. Data were collected by form which included demographic characteristics (age, parity, education, occupation) question related to current pregnancy, medical and reproductive history (history of abortion and premature labor) and detailed information about vaginal bleeding.

Vaginal bleeding was observed and classified according to our objectives such as time (no bleeding, first trimester only, second trimester only and both first and second trimesters), episodes (No, single and multiple episodes), duration (No, 1-2 days,>2 days) and severity (No, Spotting, More than menstrual period). Also case group (delivery under 37 weeks) was categorized in two groups based on gestational age, which defined as less than 34 weeks and 34-37 weeks of gestational age. Furthermore, they were divided in two groups regarding to ROM (spontaneous preterm labor and preterm premature rupture of membrane).

**Statistical analysis**

Data were express as mean, standard deviation, absolute and relative frequency and were analyzed by T-test, chi-square and, logistic regression (Forward Wald model) in SPSS version 16 software. 95% confidence interval and p-values of less than 0.05 was considered to indicate statistical significance.

## Results

Findings showed that 21.6% of women had vaginal bleeding during the first and second trimester. Results mentioned that there was significant relation between work during current pregnancy (p=0.001), previous abortion (p=0.009), and previous preterm delivery (p=0.001) with current preterm birth (Table I). Results revealed that 30.5% of case and 12.7% of control group experienced vaginal bleeding during the first and second trimester and showed that bleeding were associated significantly with 3 times increased risk of preterm delivery (p=0.0001) (OR= 3, 95% confidence interval CI: 1.84-4.89). Although bleeding in second trimester was associated with more increased risk of preterm delivery, but there were considerably weaker (Table II).

Furthermore, the relation between vaginal bleeding characteristics in groups demonstrated significant relation between multiple episodes (p=0.0001), multiple days (p=0.0001) and more blood loss (p=0.0001) with preterm birth (Table III). In additaion the relation between vaginal bleeding and preterm type (under 34 and 34-37 weeks of gestation) obtained that 69.5% of cases delivered between 34 and 37 weeks of gestation and findings revealed that women under 34 and 34-37 weeks of gestation had 43.3% and 24.8% vaginal bleeding, respectively (Table IV). Besides, women under 34 weeks experienced more multiple episodes, more than 2 days and heavier bleeding. Also, results according to preterm delivery subtypes which were defined as spontaneous preterm labor and preterm premature rupture of membrane, showed no significant relation (p=0.173) (Table V). Result regarding to preterm birth and potential risk factor in logistic regression analysis is shown in table VI.

**Table I:** Maternal demographic characteristics during pregnancy in groups

**Characteristics**		**Case (n=220)**		**Control (n=220)**		**p-value**
		**N**	**%**	**N**	**%**	
Age (years)						0.149
	<20	22	10.0	31	14.1	
	20-34	160	72.7	163	74.1	
	>35	38	17.3	26	11.8	
Education						0.064
	Less than 12 years	203	92.3	212	94.6	
	12 years and more	17	7.7	8	3.6	
Work during pregnancy		18	8.2	3	1.4	0.001
Previous abortion		55	25.0	33	15.0	0.009
Previous preterm birth		23	10.5	4	1.8	0.001
Parity						0.565
	1	119	54.1	125	56.8	
	2 and more	101	45.9	95	43.2	

* chi-square

**Table II:** Vaginal bleeding and risk of preterm delivery in groups

**Characteristics**		**Case (n=220)**		**Control (n=220)**		**p-value**
		**N**	**%**	**N**	**%**	
Vaginal bleeding						0.0001
	Yes	67	30.5	28	12.7	
	No	153	69.5	192	87.3	
Trimester of bleeding						0.0001
	No	153	69.5	192	87.3	
	First trimester only	42	19.1	23	10.5	
	Second trimester only	17	7.7	2	0.9	
	Both trimesters	8	3.6	3	1.4	

* chi-square

**Table III:** Vaginal bleeding characteristics in groups

**Characteristics**		**Case (n=220)**		**Control (n=220)**		**p-value**
		**N**	**%**	**N**	**%**	
Bleeding episode						0.0001
	No	153	69.5	192	87.3	
	Single	27	12.3	9	4.1	
	Multiple	40	18.2	19	8.6	
Duration (days)						0.0001
	No	153	69.5	192	87.3	
	1-2	33	15.0	9	4.1	
	>2	34	15.5	19	8.6	
Severity						0.0001
	No	153	69.5	192	87.3	
	Spotting	58	26.4	22	10	
	More than menstural period	9	4.1	6	2.7	

* chi-square

**Table IV:** Vaginal bleeding characteristics in preterm groups

**Characteristics**		**Under 34 weeks of gestation (n=67)**		**34-37 weeks of gestation (n=153)**		**p-value**
		**N**	**%**	**N**	**%**	
Vaginal bleeding						0.006
	Yes	29	43.3	38	24.8	
	No	38	56.7	115	75.2	
Trimester of bleeding						0.001
	No	38	56.7	115	75.2	
	First trimester only	14	20.9	28	18.3	
	Second trimester only	8	11.9	9	5.9	
	Both trimesters	7	10.4	1	0.7	
Bleeding episode						0.001
	No	38	56.7	115	75.2	
	Single	7	10.4	20	13.1	
	Multiple	22	32.8	18	11.8	
Duration (days)						0.005
	No	38	56.7	115	75.2	
	1-2	11	16.4	22	14.4	
	>2	18	26.9	16	10.5	
Severity						0.024
	No	38	56.7	115	75.2	
	Spotting	25	37.3	33	21.6	
	More than menstural period	4	6	5	3.3	

* chi-square

**Table V:** Vaginal bleeding and preterm delivery subtypes

		**Preterm delivery**	**spontaneous preterm labor** **(** **n=106)**		**Preterm (premature rupture of membrane)** **(** **n=114)**		**p-value**
**subtypes**			**N**	**%**	**N**	**%**	
Vaginal bleeding							0.173
	Yes		36	34	31	27.2	
	No		70	66	83	72.8	
Trimester of bleeding							0.526
	No		70	66	83	72.8	
	First trimester only		24	22.6	18	15.8	
	Second trimester only		9	8.5	8	7	
	Both trimesters		3	2.8	5	4.4	
Bleeding episode							0.12
	No		70	66	83	72.8	
	Single		11	10.4	16	14	
	Multiple		25	23.6	15	13.2	
Duration (days)							0.518
	No		70	66	83	72.8	
	1-2		17	16	16	14	
	>2		19	17.9	15	13.2	
Severity							0.461
	No		70	66	83	72.8	
	Spotting		32	30.2	26	22.8	
	More than menstural period		4	3.8	5	4.4	

* chi-square

**Table VI:** Preterm birth and potential risk factor in logistic regression analysis (forward wald model

		**OR**	**95% CI**	**p-value**
Job				0.001
	Housewife	1	-	
	Worker	7.67	(2.19-26.9)	
Prior abortion		1.93	(1.17-3.21)	0.01
Prior preterm birth		6.93	(2.31-20.77)	0.01
Any bleeding		3.12	(1.88-5.17)	0.01

## Discussion

This study found that vaginal bleeding was associated with 3 times increased risk of pretern delivery (OR= 3, 95% CI: 1.84-4.89). In addition our finding showed that vaginal bleeding characteristics including more severe bleeding, multiple episode and multiple days were associated with greater risk of prertm delivery, which was consistent with Yang *et al* (OR: 1.3, 95% CI: 1.1-1.6) and Hossain *et al* (OR: 1.60, 95% CI: 1.20-2.13) (1, 4). But inconsistent with Stropino *et al* and Sun *et al* which mentioned no considerable relation between heavier bleeding and preterm delivery (9, 10).

Our research also discovered notably stronger association between more severe vaginal bleeding in both the first and second trimester of pregnancy and risk of preterm delivery which was similar with another study that revealed greater risk of preterm delivery with the severity of bleeding (11). However our results showed that there were no significant relation between preterm subtypes (spontaneous rupture of membrane and premature rupture of membrane) and vaginal bleeding, but these findings were dissimilar with Yang *et al* who showed significant relation between vaginal bleeding and preterm premature rupture of membrane and Hossain *et al* which indicated that vaginal bleeding was more strongly related with spontaneous preterm labor (1, 4).

The association between vaginal bleeding and preterm delivery has also been noted by others physicians (10, 12, 13). In our study, results revealed that bleeding in second trimester was associated with more increased risk of preterm delivery, but there were considerably weaker. In which in another study the effects of vaginal bleeding during first and second trimester on pregnancy outcomes was assessed and showed that association between second trimester vaginal bleeding and preterm labour was highly significant (p<0.001) (14).

Also in another study, women with first-trimester bleeding had an increased risk of preterm labor in the same pregnancy and the proportion of women experiencing first-trimester bleeding increased by decreasing gestational age at delivery. First trimester bleeding increased the risk of preterm delivery in weeks 32-36 from 3.6-6.1% (OR: 1.65; 95% CI: 1.57-1.77) and in weeks 28-31 from 0.3-0.9% (OR: 2.98; 95% CI: 2.50-3.54). In addition, women with first-trimester bleeding had a 1.19-fold (95% CI: 1.11-1.28) increased risk of PROM and 1.18-fold (95% CI: 1.01-1.37) increased risk of preterm PROM (12).

Hossain *et al* reported that any vaginal bleeding in early pregnancy was associated with a 1.57 fold increased risk of preterm delivery (95% CI: 1.16-2.11). Vaginal bleeding was most strongly related with spontaneous preterm labor (OR: 2.10) and weakly associated with preterm premature rupture of membrane (OR: 1.36). As compared to women with no bleeding, those who bled during the first and second trimesters had a 6.24-fold increased risk of spontaneous preterm labor; and 2-3 fold increased risk of medically induced preterm delivery and preterm premature rupture of membrane, respectively (1).

Although, result showed that preterm delivery is increased significantly in patients with either light or heavy vaginal bleeding in first and second trimester of pregnancy. However in one study, the investigator failed to show a relation between preterm deliveries before 36 weeks of gestation with light vaginal bleeding in the first or second trimester of pregnancy (5). Also we found that recurrent bleeding predicted preterm delivery more strongly than did single episodes, consistent with the other studies (1, 12, 15).

Subjective estimation of symptom, vaginal bleeding and amount of blood loss were our study limitations and recall errors of bleeding characteristic, particularly under reporting early light bleeding could have happened because the bleeding characteristics such as duration and severity of vaginal bleeding were collected late in pregnancy and based on subjective description by patients. Therefore, women with higher risk of preterm delivery might report more severe vaginal bleeding because of increased anxiety. 

On the other hand, misclassification of maternal vaginal bleeding could be possible, because women may confuse menstrual bleeding with vaginal bleeding in early pregnancy. Also absence of detailed complete informations of genital tract infections during pregnancy could be another limitation. However strength of this study is the assessment of all aspects of vaginal bleeding in pregnancy in which preterm delivery and it's subtypes in relation to intensity, volume of blood loss, duration and number of episodes of vaginal bleeding was evaluated. According to significant association between vaginal bleeding and preterm delivery, it seems that performing some interventions to prevent preterm labor could be appropriate.

## References

[1] Hossain R, Harris T, Lohsoonthorn V, Williams MA (2007). Risk of preterm delivery in relation to vaginal bleeding in early pregnancy. Eur J Obstet Gynecol Reprod Biol.

[2] Mattison DR, Damus K, Fiore E, Petrini J, Alter C (2001). Preterm delivery: a public health perspective. Paediatr Perinat Epidemiol.

[3] Axelsen SM, Henriksen TB, Hedegaard M, Secher NJ (1995). Characteristics of vaginal bleeding during pregnancy. Eur J Obstet Gynecol Reprod Biol.

[4] Yang J, Hartmann KE, Savitz DA, Herring AH, Dole N, Olshan AF (2004). Vaginal bleeding during pregnancy and preterm birth. Am J Epidemiol.

[5] Weiss JL, Malone FD, Vidaver J, Ball RH, Nyberg DA, Comstock CH (2004). Threatened abortion: A risk factor for poor pregnancy outcome, a population-based screening study. Am J Obstet Gynecol.

[6] Elovitz MA, Baron J, Phillipe M (2001). The role of thrombin in preterm parturition. AM J Obstet Gynecol.

[7] Rosen T, Kuczynski E, O'Neill LM, Funai EF, Lockwood CJ (2001). Plasma levels of thrombin-antithrombin complexes predict preterm premature rupture of the fetal membranes. J Matern Fetal Med.

[8] Saraswat L, Bhattacharya S, Maheshwari A, Bhattacharya S (2010). Maternal and perinatal outcome in women with threatened miscarriage in the first trimester: a systematic review. BJOG.

[9] Strobino B, Pantel-Silverman J (1989). Gestational vaginal bleeding and pregnancy outcome. Am J Epidemiol.

[10] Sun L, Tao F, Hao J, Su P, Liu F, Xu R (2012). First trimester vaginal bleeding and adverse pregnancy outcomes among Chinese women: from a large cohort study in China. J Matern Fetal Neonatal Med.

[11] Chung TK, Sahota DS, Lau TK, Mongelli JM, Spencer JA, Haines CJ (1999). Threatened abortion: prediction of viability based on signs and symptoms. Aust N Z J Obstet Gynaecol.

[12] Lykke JA, Dideriksen KL, Lidegaard O, Langhoff-Roos J (2010). First-trimester vaginal bleeding and complications later in pregnancy. Obstet Gynecol.

[13] Yoneyama K, Kimura A, Kogo M, Kiuchi Y, Morimoto T, Okai T (2009). Clinical predictive factors for preterm birth in women with threatened preterm labour or preterm premature ruptured membranes?. Aust N Z J Obstet Gynaecol.

[14] Karim SA, Bakhtawar I, Butta AT, Jalil M (1998). Effects of first and second trimester vaginal bleeding on pregnancy outcome. J Pak Med Assoc.

[15] Jouppila P (1979). Vaginal bleeding in the last two trimesters of pregnancy. A clinical and ultrasonic study. Acta Obstet Gynecol Scand.

